# Deciphering the Role of Reshaped Fungal Microbiome in Cadmium Accumulation in Rice Grains

**DOI:** 10.3390/jof11120837

**Published:** 2025-11-27

**Authors:** Weijun Gong, Minghui Chen, Yibin Lai, Dian Yang, Marcos Antônio Soares, Surendra Kumar Gond, Haiyan Li

**Affiliations:** 1 Life Science and Technology & Medical Faculty, Kunming University of Science and Technology, Kunming 650500, China; weijung98@126.com (W.G.); chenminghui2153122@126.com (M.C.); laiyibin2839@163.com (Y.L.); ydian9126@163.com (D.Y.); 2 Department of Botany and Ecology, Federal University of Mato Grosso, Cuiabá 78060-900, MT, Brazil; drmasoares@gmail.com; 3 Department of Botany, MMV, Banaras Hindu University, Varanasi 221005, India; surendragond@gmail.com

**Keywords:** food security, microbiome reshaping, rhizospheric community, rice grains cadmium concentration, seed endophytic fungi

## Abstract

Rice cadmium (Cd) contamination is a serious threat to global food security and human health. Plant-associated microbiomes are known to affect Cd accumulation in plants. However, the response of the rice microbiome to Cd contamination and its role in modulating grain Cd accumulation remain poorly understood. In the present study, the responses of the rhizospheric fungi (RF) community and seed endophytic fungi (SEF) community to the soil physiochemical properties of rice from moderately (MC) and severely (SC1 and SC2) Cd-contaminated paddies were investigated. Moreover, the effects of soil physiochemical properties, RF community and SEF community on grain Cd accumulation were analyzed through correlation analysis. The results showed that the Cd concentration in rice grains from SC2 exceeded the food safety standard of China and was higher than that of SC1 and MC. The Cd concentration in rice grains was positively correlated with the soil-available Cd concentration, while being negatively correlated with the available nutrient elements and pH value of soil. In addition, it was found that the diversity of RF increased with the soil-available Cd concentration, while the diversity and richness of SEF decreased with the soil-available Cd concentration. Moreover, the RF community was influenced by soil physiochemical properties. The Spearman correlation analysis showed that the soil-available Cd was positively correlated with RF *Sebacina*, *Clonostachys*, *Acremonium*, *Talaromyces* and *Fusarium*, and most of them were related to grain Cd concentration, while unclassified SEF Pleosporales and Xylariales were associated with grain Cd concentration. These results suggested that Cd stress triggered a niche-specific response of the rice fungal microbiome. The fungi related to soil Cd availability and rice grain Cd accumulation may have a great potential application in food safety production in Cd-contaminated soil.

## 1. Introduction

The contamination of soil with heavy metals (HMs) has become a global environmental problem because of the rapid development of industry and agriculture [1,2]. As one of the most toxic contaminants, cadmium (Cd) is a non-biodegradable HM widely found in agricultural soil [3]. Yuan et al. [4] reported that approximately 33.5% of farmland soil in China was contaminated with Cd. Since the latter has a great fluidity and is easily enriched in plants, soil with accumulated Cd will cause plant roots to become shorter, leaves to become yellow and even plant deaths due to its adverse effects on the physiological and biochemical processes in plants, such as nutrient transport, nitrogen metabolism, photosynthesis, etc. [5,6,7]. Moreover, Cd can enter the food chain through crops and eventually accumulate in the human body, causing great harm to the heart, bones, kidneys, brain and other organs [8,9,10]. Therefore, it is important to remediate Cd-contaminated soil and promote safe crop production.

The rhizospheric microbiome is considered as the second genome of plants, and their role in assisting host plants to enhance nutrient uptake, abiotic and biotic stress resistance has been widely demonstrated [11,12]. Currently, with the extensive contamination of HMs in the soil worldwide, a growing number of studies have reported interactions between rhizospheric microbes and host plants to resist contamination stress. For example, He et al. [13] found that rare microbes in rhizospheric soil displayed a significant role in plant Cd uptake, while the phytoextraction efficiency of Cd by *Lolium multiflorum* L. decreased when rare microbes decreased. As an important part of the rhizospheric microbiome, fungi such as *Aspergillus*, *Trichoderma* and others can regulate plants’ Cd accumulation and enhance their growth by producing organic acid and phytohormones, solubilizing insoluble nutrient elements and improving the antioxidant defense system of plants [14,15]. In addition, various additives (biochar, synthetic bacterium, nanoparticles, etc.) can improve plants’ HM resistance and regulate their HM uptake by affecting the rhizospheric microbiome [16,17,18].

Recently, the use of endophytes has attracted the attention of researchers. Previous research had demonstrated that many endophytes were resistant to HMs, were able to improve plant growth and altered plants’ HM accumulation [19,20]. For instance, Lin et al. [21] found that endophytic fungi *Serendipita indica* enhanced Cd and phosphorus (P) bioavailability and enzyme activity in soil by degrading organic matter and acidifying rhizosphere soil, thereby increasing the biomass and Cd accumulation of *Salix suchowensis*. In particular, seed endophytes can vertically transmit and become the first microbes to colonize a plant, and they may play a crucial role in the health, productivity and phytoextraction efficiency of plants under HM stress [22,23]. Truyens et al. [24] reported that seed endophytes from *Agrostis capillaris* improved its growth and Cd uptake.

Rice (*Oryza sativa*) is a staple food for at least half of the world’s population but is particularly susceptible to Cd uptake from contaminated soil [25]. Unfortunately, 5% of the global polished rice and nearly 10% of the polished rice from China exceeded the Cd standards for rice grains of Europe and China (0.2 mg/kg) [26,27]. Numerous studies have demonstrated that Cd transferability into rice grains depends on soil characteristics and microbiome and plant features [28,29]. However, there is little known about the responses of the rhizospheric fungal community and seed endophytic fungal community recruited in rice for Cd contamination and their role in Cd accumulation in rice grains. In the present study, the responses of the rhizospheric fungi (RF) community and seed endophytic fungi (SEF) community to soil properties were investigated. Moreover, the effects of soil properties, RF community and SEF community on grain Cd accumulation were revealed through correlation analysis.

This study specifically proposed the following hypotheses: (i) the RF community and SEF community of rice in paddies are driven by environmental factors, especially Cd concentration; (ii) there are some specific fungi which can regulate Cd accumulation in rice grains, and they can be explored in safe rice production. By systematically testing these hypotheses, this research aims to provide new insights into the rice–fungi interaction and offer potential fungal targets in food safety.

## 2. Materials and Methods

### 2.1. Sample Collection

The soil and rice seeds were collected from moderately (MC, containing 2.00 mg/kg total Cd) and severely (SC1 and SC2, containing 3.29 and 2.93 mg/kg total Cd, respectively) Cd-contaminated paddies near mines. Site MC was located in Zhehai Town, Huize County, Yunnan Province (103°34′39″ E, 26°30′50″ N); site SC1 was located in Wanshan Town, Wanshan District, Guizhou Province (109°11′49″ E, 27°30′49″ N); and site SC2 was located in Shuikoushan Town, Changning County, Hunan Province (112°37′24″ E, 26°33′29″ N). The MC soil with a pH of 6.72 contained 82.19 mg/kg organic matter (OM), 240.03 mg/kg hydrolyzable nitrogen (HN), 37.10 mg/kg available phosphorus (AP), 137.00 mg/kg available potassium (AK) and 1.26 mg/kg available Cd. The SC1 soil with a pH of 6.81 contained 43.96 mg/kg OM, 181.10 mg/kg HN, 20.40 mg/kg AP, 116.50 mg/kg AK and 2.12 mg/kg available Cd. The SC2 soil with a pH of 4.68 contained 45.27 mg/kg OM, 166.55 mg/kg HN, 4.75 mg/kg AP, 89.75 mg/kg AK and 2.73 mg/kg available Cd.

Sampling was performed in September 2023. At each site, rhizospheric soil and grains (seeds) of rice were collected using the five points method, and about 100 g of rhizospheric soil was collected as previously described [30]. Briefly, rice was collected from fields during the maturity stage; then, soil was shaken off, and rhizospheric fractions were brushed and collected for further processing. The five sub-samples were combined and then separated into four replicates. Some rice grains were husked and then surface-sterilized according to the previous method [31]. Briefly, rice grains were sterilized through immersion in 75% (*v*/*v*) ethanol for 2 min and washed three times with sterile water, followed by immersion in 5% sodium hypochlorite for 1 min and washing five times with sterile water. One part of rice rhizospheric soil and surface-sterilized grains was immediately frozen in liquid nitrogen and stored at −80 °C for subsequent microbial DNA extraction, and the other was air-dried (25 °C) for physicochemical property and Cd concentration analysis.

### 2.2. Soil Physicochemical Property and Grain Cd Concentration Analysis

After being air-dried, the soil was crushed and sieved with a 0.15 mm mesh to obtain fine powders. The soil OM was determined using potassium dichromate (K_2_Cr_2_O_7_) and sulfuric acid (H_2_SO_4_) oxidation and titration [32]. The soil HN, AP and AK were determined through alkaline hydrolysis, sodium bicarbonate leaching–molybdenum antimony colorimetry and ammonium acetate extraction–atomic absorption spectrometry methods, respectively [33]. Soil pH was measured after shaking for 0.5 h at a water–soil ratio of 2.5:1 [32]. To determine the total Cd concentration in the soil, soil powders (0.5 g) were digested with a 4 mL HCl–HNO_3_ (3:1, *v*/*v*) mixture at 80 °C for 30 min, then at 100 °C for 30 min and finally at 120 °C for 1 h. Thereafter, the samples were cooled, and 1 mL HClO_4_ was added to continue digesting at 100 °C for 20 min, followed by 120 °C for 1 h. The available Cd in the soil was extracted with diethylene diethylenetriaminepentaacetic acid (DTPA). Briefly, 5 g of air-dried soil was added to 25 mL of DTPA at 180 r/min and 25 °C for 2 h, and then suspensions were separated through filtration with a 0.22 mm filter. To determine the total Cd concentration in grains, the samples (0.5 g) were digested in a HNO_3_–HClO_4_ (9:1, *v*/*v*) mixture at 120 °C for 10 min, followed by 150 °C for 30 min and then 200 °C for 120 min on an electric hot plate. Then, the Cd concentrations were determined using flame atomic absorption spectrometry (ZEEnit^®^ 700P, Analytik Jena AG, Jena, Germany) [34,35].

### 2.3. DNA Extraction and Sequencing of Rice Rhizospheric Fungi and Seed Endophytic Fungi

Fungus DNA in rhizosphere soil and the seeds of rice was extracted using the FastDNA Spin Kit (MP Biomedicals, Santa Ana, CA, USA) following the manufacturer’s protocol [36]. The fungal internal transcribed spacer-1 (ITS1) region was amplified using universal primers ITS1F/ITS2R [37]. The volume of the PCR reaction was 25 μL, containing 12.5 μL 2× Taq PCR Master Mix (Takara Bio Inc., Shiga, Japan), 1 μL of each DNA sample, 1 μL of each primer and 9.5 μL double distilled water. The PCR reaction was a pre-denaturation at 95 °C for 5 min, 30 cycles of 95 °C for 30 s, 53 °C for 30 s and 72 °C for 30 s and a final extension at 72 °C for 10 min [38,39]. Every PCR was also accompanied by a negative control to ensure that the barcodes and master mix were not contaminated. The PCR products were purified with an OMEGA Gel Extraction Kit (Omega Bio-Tek, Norcross, GA, USA) according to the manufacturer’s protocol. The resulting amplicons were subsequently subjected to high-throughput sequencing using the Illumina Nextseq 2000 platform from Shanghai Majorbio Bio-pharm Technology Company (Shanghai, China).

### 2.4. Data Processing of High-Throughput Sequencing

Forward and reverse sequences were merged by overlapping paired-end reads using FLASH (v1.2.11, https://ccb.jhu.edu/software/FLASH/index.shtml, accessed on 16 July 2025). All sequence reads with the same tag were assigned to the same sample according to the unique barcodes (raw tags). The raw tags were further strictly filtered through previous methods (clean tags), and the quality of clean tags was detected using Qiime (v1.9.1, http://qiime.org/install/index.html, accessed on 16 July 2025). The chimeric sequences were detected and removed using the UCHIME algorithm (http://www.drive5.com/usearch/manual/uchime_algo.html, accessed on 16 July 2025) [40]. The effective sequences were then assigned to operational taxonomic units (OTUs) at 97% sequence similarity using the UPARSE software package (v11, http://www.drive5.com/uparse/, accessed on 16 July 2025), based on which the diversity within (alpha diversity) and between samples (beta diversity) was analyzed using the Mothur software (v1.30.2, https://www.mothur.org/wiki/Download_mothur, accessed on 16 July 2025) and Qiime [37]. Finally, a representative sequence for fungal OTU was taxonomically classified and annotated according to the UNITE database (Release 8, https://unite.ut.ee/, accessed on 16 July 2025) [38]. The OTUs aligned to chloroplast and mitochondria sequences were removed to avoid host plant gene interference.

The microbial community distribution and dissimilarity were investigated through principal coordinate analysis (PCoA) based on the Bray–Curtis distance. An analysis of similarity (ANOSIM) based on the Bray–Curtis distance was used to examine whether there were significant differences in the community structures of rice fungi in different sites. PCoA and ANOSIM were processed with the “Vegan” package in R software version 4.4.3 [41]. Linear discriminant analysis (LDA) effect size (LEfSe) biomarker analysis was utilized to explore the dissimilarities in the species composition among the samples, and an LDA score of 3 was applied as a threshold (*p* < 0.05). Redundancy analysis/canonical correlation analysis (RDA/CCA) was used to examine the effects of environmental factors on the rhizospheric fungal community and seed endophytic fungal community. The use of RDA or CCA depended on the length of the longest gradient in the previously conducted detrended correspondence analysis (DCA), and, if it was greater than 4.0, CCA was used; otherwise, RDA was used [42,43].

### 2.5. Statistical Analysis

Statistical analysis was conducted using IBM SPSS statistics 23 and R (v4.4.3). One-way ANOVA (analysis of variance) and least significant difference (LSD) tests were used to estimate the significance of differences in soil physiochemical properties, soil and grain Cd concentrations in different sites. The Kruskal–Wallis rank-sum test was performed to determine the differences in relative abundance of fungal genera and alpha diversity indexes. A Pearson correlation analysis was used to investigate the interaction effects of soil physiochemical properties, soil and grain Cd concentrations. The influence of environmental factors on the diversity and dominant genera of fungal communities was analyzed through the Spearman correlation analysis. The environment factor data were presented as mean ± standard deviation (SD), while fungal alpha diversity indexes were presented as quartiles.

## 3. Results

### 3.1. The Key Environmental Factors Affecting Cd Accumulation in Rice Grains

There were significant differences in the soil physiochemical properties and Cd concentration in rice grains among different sites (*p* < 0.05) (Table 1). The pH values of the soil from sites MC and SC1 were neutral (6.72 and 6.81), while the pH value of the soil from site SC2 was acidic (4.68) and significantly lower than those of sites MC and SC1 (*p* < 0.05). The soil OM at site MC was higher than that of sites SC1 and SC2 (*p* < 0.05), but there was no significant difference in the soil OM between site SC1 and site SC2 (*p* > 0.05). The soil HN, AP and AK followed the order of site MC > site SC1 > site SC2 (*p* < 0.05). Compared with site MC, the total Cd concentration in the soil from site SC1 and site SC2 increased by 64.50% and 46.50%, respectively, and the available Cd concentration in the soil increased by 68.25% and 116.67%, respectively (*p* < 0.05). Notably, the total Cd concentration in the soil from sites SC1 and SC2 exceeded the risk intervention values in soil for China, which indicated that the soil was severely contaminated with Cd and the risk to agricultural product safety was high. However, the total Cd concentration in the soil from site MC was less than the risk intervention values in soil for China and was moderately Cd-contaminated [44]. Moreover, the Cd concentration in rice grains at site SC2 exceeded the maximum allowable concentration (MAC) in rice grains for China [45] and was 19.85 and 16.13 times higher than that of sites MC and SC1, respectively.

**Figure 1:**
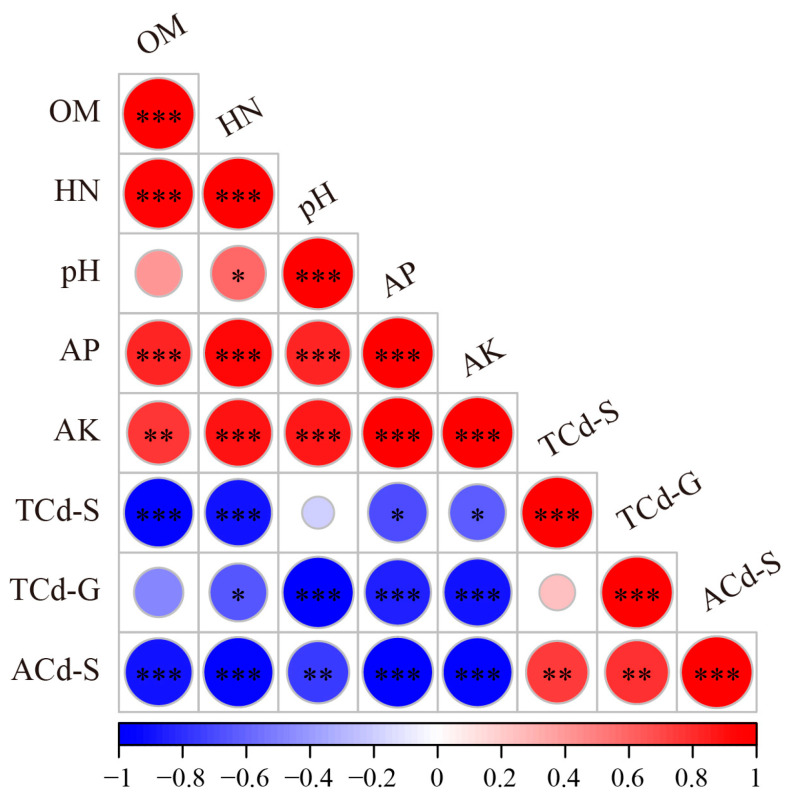
Correlations between the soil physiochemical properties and Cd concentration in rice grains. OM: organic matter; HN: hydrolyzable nitrogen; AP: available phosphorus; AK: available potassium; TCd-S: total cadmium concentration in soil; ACd-S: available cadmium concentration in soil; TCd-G: total cadmium concentration in rice grains. The “*” indicates statistically significant difference (*, *p* < 0.05; **, *p* < 0.01; ***, *p* < 0.001).

To determine the key environmental factors affecting Cd accumulation in rice grains, a Pearson correlation analysis was conducted between grain Cd concentrations and soil physiochemical properties (Figure 1). The results showed that the grain Cd concentration was significantly positively correlated with the available Cd concentration in the soil (*r* = 0.796, *p* < 0.01). Meanwhile, the grain Cd concentration was significantly negatively correlated with soil HN (*r* = −0.651, *p* < 0.05), pH (*r* = −0.986, *p* < 0.001), AP (*r* = −0.856, *p* < 0.001) and AK (*r* = −0.899, *p* < 0.001).

### 3.2. Effect of Cd Stress on Community Composition of Rice RF and SEF

**Figure 2:**
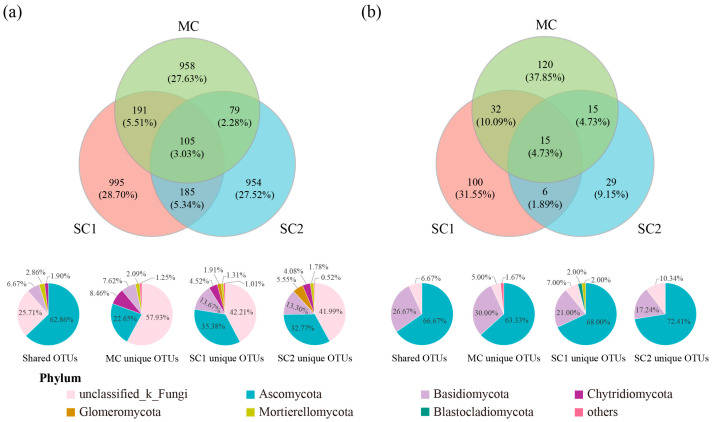
The unique and shared fungal OTUs of rhizospheric soil (**a**) and seeds (**b**) for rice from different cadmium-contaminated sites. Pie charts represent composition of shared and unique OTUs at the phylum level. The fungi with a percentage below 1% were grouped as “others”. Percentages may not total 100% due to rounding. MC represents the moderately cadmium-contaminated site; SC1 and SC2 represent the severely cadmium-contaminated sites.

After quality filters, 1,015,975 and 862,752 valid sequence reads were recovered from 12 rhizospheric soils and 12 rice seeds, respectively. The reads of RF for rice were clustered in 3467 OTUs, and 105 (3.03%) OTUs were shared by sites MC, SC1 and SC2, while these OTUs were dominated by Ascomycota (62.86%) and unclassified fungi (25.71%). Notably, 958 (27.63%), 995 (28.70%) and 954 (27.52%) fungal OTUs were only detected in rhizospheric soil from sites MC, SC1 and SC2, respectively, while unclassified fungi (>41.99%) were most dominant in these OTUs (Figure 2a). In addition, the reads of SEF for rice were clustered in 317 OTUs, while 15 (4.73%) OTUs were shared by sites MC, SC1 and SC2. Moreover, 120 (37.85%), 100 (31.55%) and 29 (9.15%) endophytic fungal OTUs were only detected in rice seeds from sites MC, SC1 and SC2, respectively. Ascomycota and Basidiomycota were the dominant taxa in the shared and unique SEF OTUs (Figure 2b).

**Figure 3:**
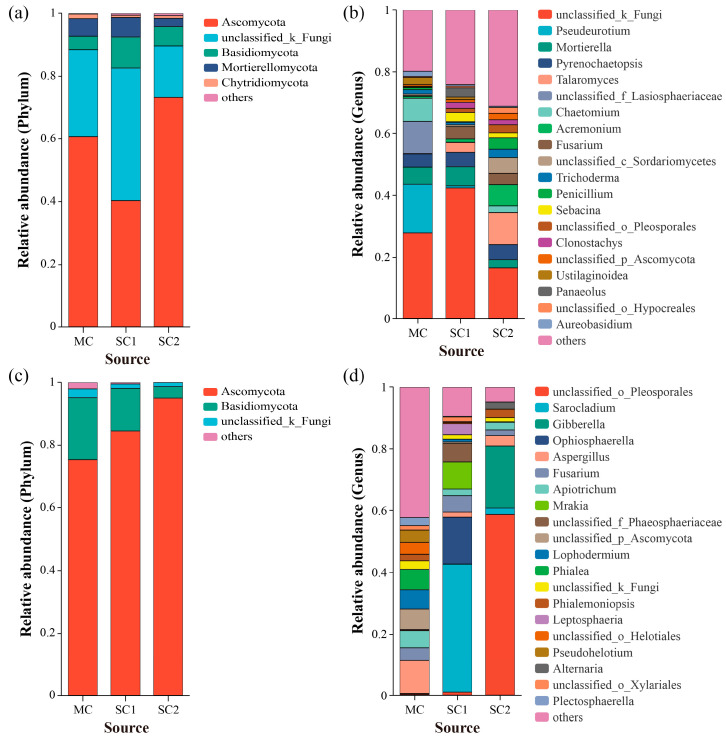
Fungal community composition and relative abundance of rhizospheric soil and seeds for rice from different cadmium-contaminated sites. (**a**,**b**) Composition and relative abundance of rhizospheric fungi at the phylum and genus level, respectively. (**c**,**d**) Composition and relative abundance of seed endophytic fungi at the phylum and genus level, respectively. MC represents the moderately cadmium-contaminated site; SC1 and SC2 represent the severely cadmium-contaminated sites.

The RF OTUs were identified to 12 phyla, 40 classes, 102 orders, 239 families and 479 genera. At the phylum level, Ascomycota was the most dominant taxon at sites MC (60.71%) and SC2 (73.17%), while unclassified fungi (42.30%) were the most dominant taxon at site SC1 (Figure 3a). At the genus level, unclassified fungi displayed the most relative abundance at sites MC (27.74%), SC1 (42.30%) and SC2 (16.47%). Other dominant genera from site MC were *Pseudeurotium* (15.75%), unclassified Lasiosphaeriaceae (10.44%), *Chaetomium* (7.47%), *Mortierella* (5.58%) and *Pyrenochaetopsis* (4.27%). However, after the unclassified fungi, the RF from site SC1 were dominated by *Mortierella* (6.21%), *Pyrenochaetopsis* (4.65%), *Fusarium* (3.95%), *Talaromyces* (3.19%) and *Panaeolus* (3.04%). At site SC2, the dominant RF *Talaromyces* (10.38%), *Acremonium* (6.84%), unclassified Sordariomycetes (5.10%), *Pyrenochaetopsis* (4.89%), *Penicillium* (3.77%) and *Fusarium* (3.70%) followed the unclassified fungi (Figure 3b).

In addition, the OTUs of SEF for rice were identified to 6 phyla, 22 classes, 59 orders, 118 families and 160 genera. At the phylum level, Ascomycota was the most dominant taxon at sites MC (75.35%), SC1 (84.50%) and SC2 (95.02%), followed by Basidiomycota (Figure 3c). The relative abundance of Ascomycota in seeds increased with grain Cd concentration, but the opposite was observed for Basidiomycota. At the genus level, at site MC, *Aspergillus* possessed the most relative abundance, with 10.75%, followed by unclassified Ascomycota (6.70%), *Phialea* (6.62%), *Lophodermium* (6.20%), *Apiotrichum* (5.52%), *Fusarium* (4.14%), *Pseudohelotium* (3.98%) and unclassified Helotiales (3.82%). Furthermore, the dominant SEF at site SC1 were *Sarocladium* (41.55%), *Ophiosphaerella* (15.20%), *Mrakia* (8.80%), unclassified Phaeosphaeriaceae (6.02%), *Fusarium* (5.28%) and *Leptosphaeria* (3.57%). Meanwhile, it was found that the SEF from site SC2 were dominated by unclassified Pleosporales (58.73%), followed by *Gibberella* (20.06%) and *Aspergillus* (3.45%) (Figure 3d).

### 3.3. The Key Environmental Factors Affecting Diversity of Rice RF and SEF

**Figure 4:**
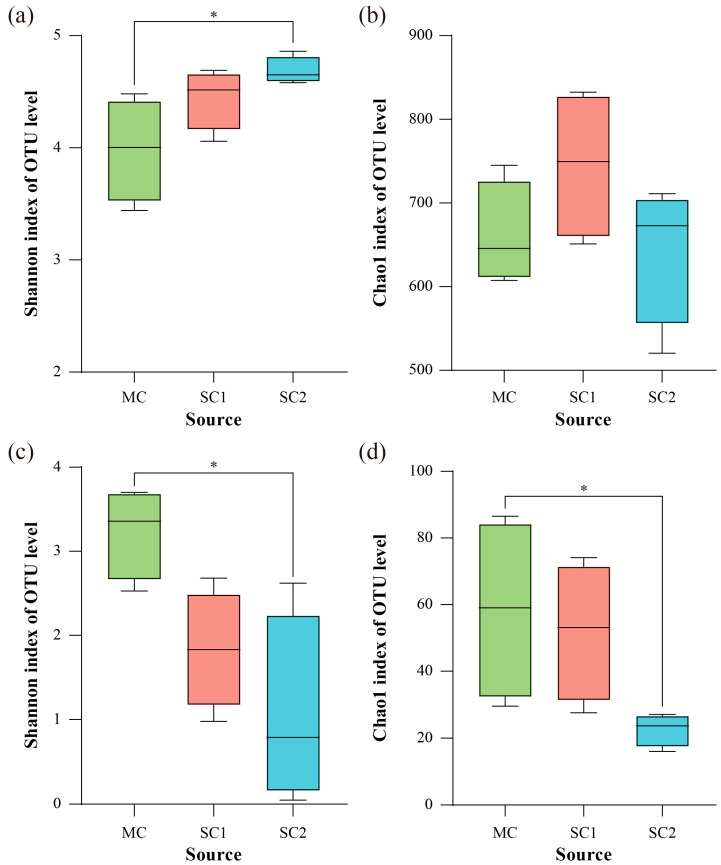
The alpha diversity of rhizospheric fungi and seed endophytic fungi for rice from different cadmium-contaminated sites. (**a**,**b**) Shannon and Chao1 indexes of rhizospheric fungi for rice. (**c**,**d**) Shannon and Chao1 indexes of seed endophytic fungi for rice. *: *p* < 0.05. MC represents the moderately cadmium-contaminated site; SC1 and SC2 represent the severely cadmium-contaminated sites.

The alpha diversity indexes of RF and SEF for rice were calculated, and the species diversity and richness were indicated by the Shannon and Chao1 indexes, respectively (Figure 4). The results showed that the species diversity of rice RF was influenced by the nutrients, pH value and available Cd concentration in soil, while only the species diversity of the rice RF from site SC2 was significantly higher than that of site MC (*p* < 0.05) (Figure 4a, Table 2). However, the species richness of rice RF from different sites showed no significant difference (*p* > 0.05) (Figure 4b). Moreover, the soil-available Cd concentration was the most dominant factor influencing the alpha diversity of SEF for rice (Table 2). The species diversity and richness of SEF decreased with an increasing soil-available Cd concentration, and there were significant differences between sites MC and SC2 (*p* < 0.05) (Figure 4c,d).

**Figure 5:**
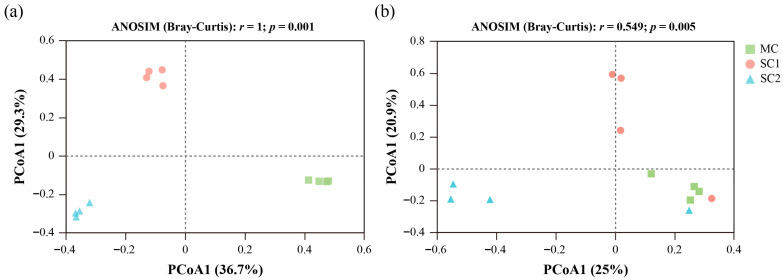
Principal coordinates analysis (PCoA) of rhizospheric fungi (**a**) and seed endophytic fungi (**b**) for rice from different cadmium-contaminated sites. MC represents the moderately cadmium-contaminated site; SC1 and SC2 represent the severely cadmium-contaminated sites.

Beta diversity was analyzed at the OTU level, and the structures of fungal communities at different sites were compared (Figure 5). The PCoA and ANOSIM based on the Bray–Curtis distance matrix showed significant differences in the rhizospheric fungal community distribution among the three sites (*r* = 1.000, *p* = 0.001), and two principal coordinates PCoA1 and PCoA2 together explained 66.0% of the total variation in the rhizospheric fungal community structure (Figure 5a). In addition, the community structures of SEF for rice from different Cd-contaminated soils showed significant differences (*r* = 0.549, *p* = 0.005), while two principal coordinates PCoA1 and PCoA2 together explained 45.9% of the total variation in the community structures (Figure 5b).

### 3.4. Biomarkers of RF and SEF for Rice from Different Cd Contaminated Sites

**Figure 6:**
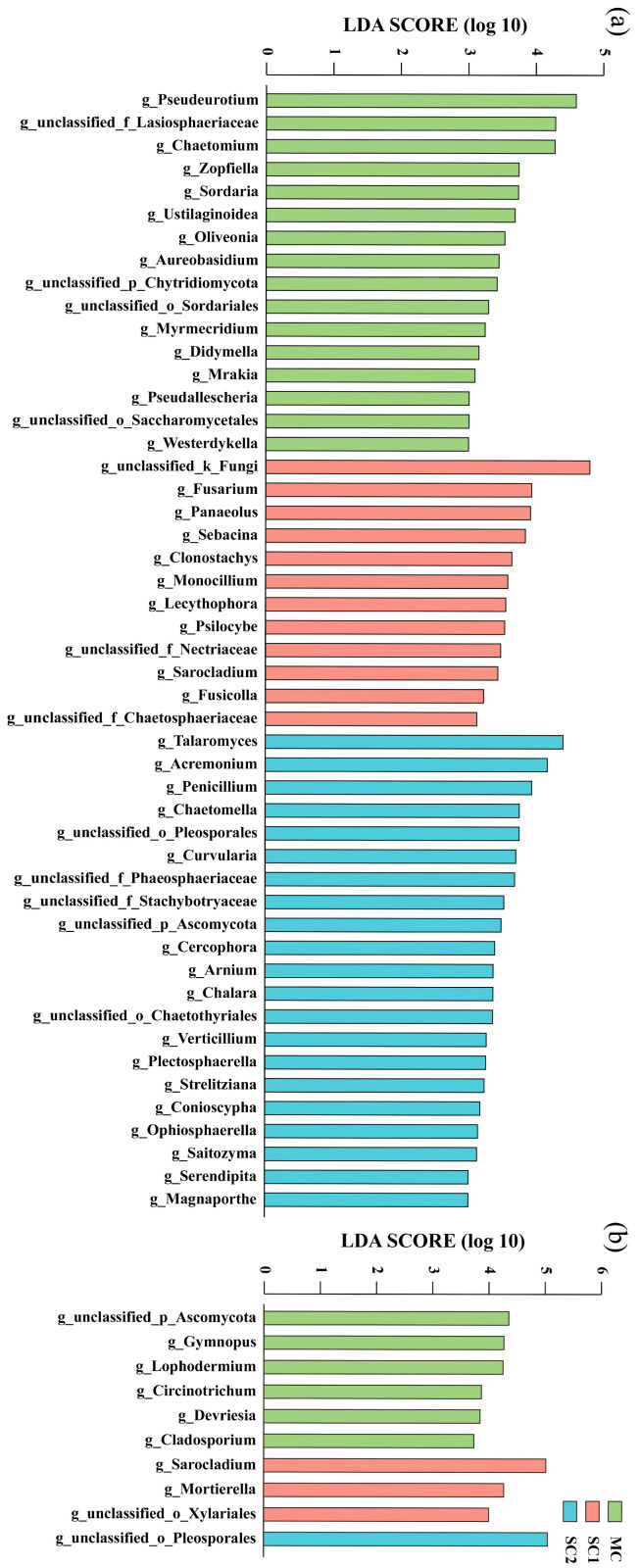
Biomarkers of rhizospheric fungi (**a**) and seed endophytic fungi (**b**) for rice from different cadmium-contaminated sites. MC represents the moderately cadmium-contaminated site; SC1 and SC2 represent the severely cadmium-contaminated sites.

To further identify key fungal genera of rice Cd tolerance and grain Cd uptake, an LEfSe analysis was used to compare the differences in rice fungi between different Cd-contaminated sites. The genera whose relative abundance significantly increased and significantly affected the differences in fungal communities were identified as biomarkers (Figure 6). In total, 16, 12 and 21 rhizospheric fungal genera were identified as biomarkers at sites MC, SC1 and SC2, respectively. At site MC, *Pseudeurotium*, unclassified Lasiosphaeriaceae, *Chaetomium*, *Zopfiella*, *Sordaria*, *Ustilaginoidea*, etc., were the rhizospheric fungal biomarkers. At site SC1, unclassified fungi, *Fusarium*, *Panaeolus*, *Sebacina*, *Bionectriaceae* and others were the rhizospheric fungal biomarkers. In addition, *Talaromyces*, *Acremonium*, *Penicillium*, *Aspergillaceae*, *Chaetomella*, etc., were the rhizospheric fungal biomarkers at site SC2. Notably, some dominant genera (relative abundance > 3%) displayed a greater influence (LDA score > 4.0) on the differences in rhizospheric fungal communities, including *Pseudeurotium*, unclassified Lasiosphaeriaceae and *Chaetomium* at site MC, unclassified fungi in SC1, as well as *Talaromyces* and *Acremonium* at site SC2 (Figure 6a).

In addition, six, three and one genera were identified as biomarkers of SEF at sites MC, SC1 and SC2, respectively. At site MC, unclassified Ascomycota, *Gymnopus*, *Lophodermium*, *Circinotrichum*, *Devriesia* and *Cladosporium* were biomarkers of SEF. Moreover, *Sarocladium*, *Mortierella* and unclassified Xylariales were biomarkers of SEF at site SC1. Meanwhile, at site SC2, unclassified Pleosporales was the only biomarker of SEF (Figure 6b).

### 3.5. The Correlations Between Fungal Communities and Environmental Factors

**Figure 7:**
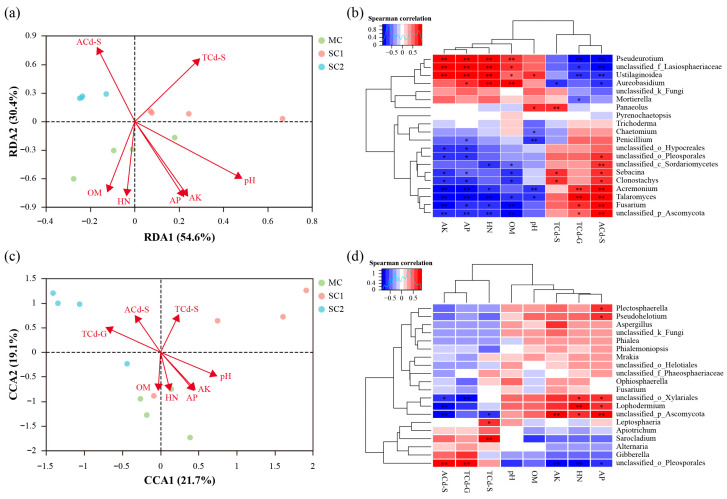
Correlations between rhizospheric fungi community (**a**) and genera (**b**), seed endophytic fungi community (**c**) and genera (**d**) and the environment factors. OM: organic matter; HN: hydrolyzable nitrogen; AP: available phosphorus; AK: available potassium; TCd-S: total cadmium concentration in soil; ACd-S: available cadmium concentration in soil; TCd-G: total cadmium concentration in rice grains. MC represents the moderate cadmium contamination site, SC1 and SC2 represent the severe cadmium contamination sites. The “*” indicates statistically significant difference (*, *p* < 0.05; **, *p* < 0.01). Only the top 20 abundant genera are shown in this figure.

The contributions of grain Cd concentrations and soil physiochemical properties to fungal communities were analyzed using RDA/CCA, and the top 20 fungi genera related to rice Cd tolerance and grain Cd accumulation were analyzed through Spearman correlation analysis (Figure 7). The results showed that soil nutrients (OM, *r*^2^ = 0.7506; HN, *r*^2^ = 0.8145; AP, *r*^2^ = 0.8989; AK, *r*^2^ = 0.9012) were the key physiochemical factor influencing the RF community of rice (*p* < 0.01) (Figure 7a, Table 3). In addition, the RF community also changed in response to Cd contamination, and the soil-available Cd concentration showed a greater contribution to variations in the community structure of RF (*r*^2^ = 0.8521, *p* = 0.002) (Figure 7a, Table 3). Moreover, the soil-available Cd concentration was positively associated with unclassified Pleosporales, unclassified Sordariomycetes, *Sebacina*, *Clonostachys*, *Acremonium*, *Talaromyces*, *Fusarium* and unclassified Ascomycota, while being negatively associated with *Pseudeurotium*, unclassified Lasiosphaeriaceae, *Ustilaginoidea* and *Aureobasidium*. At the same time, most of these taxa were correlated with the Cd concentration in rice grains (Figure 7b).

This study demonstrated that soil pH (*r*^2^ = 0.7708, *p* < 0.01) was the most dominant physiochemical factor influencing the SEF community of rice. Furthermore, the TCd-G (*r*^2^ = 0.7604, *p* < 0.01) was the main fraction affecting the endophytic fungi community of rice seeds (Figure 7c, Table 3). The TCd-G was positively associated with unclassified Pleosporales but negatively associated with unclassified Xylariales (Figure 7d).

## 4. Discussion

### 4.1. Impact of Soil Physiochemical Properties on Rice Grain Cd Accumulation

It has been found that Cd accumulation in plants was influenced by soil nutrients [46,47]. In the present study, the grain Cd concentration was significantly negatively correlated with soil-available N, P and K (Figure 1). The previous studies found that higher soil nutrients reduced plants’ Cd accumulation and translocation by reducing the soil Cd bioavailability, regulating the expressions of Cd transporters in plants, etc. For example, Shao et al. [48] revealed that activated P rocks significantly diminished rice grain Cd accumulation by decreasing the exchangeable Cd in soil and increasing Fe-Mn oxide-bound Cd and residual Cd. Moreover, Huang et al. [49] found that K application could decrease the uptake and translocation of Cd in *Ipomoea batatas* (L.) Lam by regulating the expression of genes associated with Cd transporters and root cell wall components. However, the other studies demonstrated that the increase in soil nutrients enhanced Cd accumulation and translocation in plants [47,50]. Based on these results, when fertilizers are applied to Cd-contaminated soil, both types and doses should be carefully considered to mitigate Cd accumulation in rice, especially in grains.

In addition, it was found that the grain Cd concentration had a significantly negative correlation with the soil pH (*r* = −0.986, *p* < 0.001) (Figure 1). It has been found that, among the soil characteristics that affected Cd accumulation in plants, soil pH was considered to be a crucial factor, since soil Cd bioavailability increased as soil pH decreased [28,51,52]. Similarly, in the present study, the soil-available Cd concentration was significantly negatively correlated with soil pH, while the grain Cd concentration was significantly positively correlated with the soil-available Cd concentration (Figure 1). The results indicate that reducing rice Cd accumulation by enhancing soil pH is an efficient strategy.

### 4.2. Impact of Soil Physiochemical Properties on Rice Rhizospheric Fungi Community and Its Potential Role in Rice Grain Cd Accumulation

The rhizospheric microbiome was influenced by various soil variables, which played an important role in plant stress resistance [12,53,54]. In this study, although the measured soil parameters were not significantly related to the RF species richness (Chao1 index), they were correlated with the fungi species diversity (Shannon index) (Table 2). In particular, the species diversity of RF was negatively associated with the soil pH (*r* = −0.5378, *p* < 0.05), and the acidic site SC2 displayed the highest Shannon index of RF (Figure 4a, Table 2). Contrary to this, it is generally reported that the diversity of soil microbes is higher in neutral soil but lower in acidic and alkaline soil [55,56,57]. This might be due to the combined effect of various soil factors. In the present study, the soil nutrients (OM, HN, AP and AK) were negatively associated with the species diversity of RF (*p* < 0.05) (Table 2). Moreover, the RDA showed that soil nutrients were the key physiochemical factors influencing the RF community composition of rice (*p* < 0.01) (Figure 7a, Table 3). It has been found that excessive inputs of nutrients into soil could reduce the microbe diversity of rice. For example, Sun et al. [58] reported that a low concentration of P increased fungi diversity in the rice rhizosphere. This may have occurred because P/K-loving microbes, such as *Talaromyces*, *Aspergillus*, *Fusarium* and *Epicoccum*, dominated in high K/P soil, and these were dominant in the microbial community, leading to a decreased microbial diversity [59,60,61]. These microbes may affect the solubility and availability of soil nutrients and thus influence the structure of the rhizospheric microbiome. Furthermore, the reshaped microbiome may alter the soil habitat through various biochemical and biophysical mechanisms, thereby affecting HM tolerance and accumulation in plants [62,63].

This study showed that the total and available Cd concentrations in soil significantly affected the community of rice RF (*p* < 0.01) (Figure 7a, Table 3). It has been found that HM concentration was dominant factor for shaping soil microbe communities [64]. Although multiple studies illustrated that a higher HM concentration generally decreased the microbial community diversity in soil, these studies mainly focused on the total HM concentration in soil and rarely considered the more toxic available HM concentration [65,66,67]. Meanwhile, the species diversity of RF was positively associated with the soil-available Cd concentration in this research (*r* = 0.7391, *p* < 0.05) (Table 2). This unexpected finding may be attributed to the heightened sensitivity and enhanced tolerance of RF to HM stress, which enables them to respond more rapidly to Cd stress and facilitates the emergence of fungal species with a greater tolerance to Cd, ultimately enhancing fungi diversity [68,69]. Similarly, in this study, more fungi genera were found to be enriched in rice rhizospheric soil from site SC2, with the highest available Cd concentration (Figure 6a).

In addition, the Spearman correlation analysis indicated that the soil-available Cd concentration was positively associated with *Sebacina*, *Clonostachys*, *Acremonium*, *Talaromyces* and *Fusarium*, while being negatively associated with *Pseudeurotium*, *Ustilaginoidea* and *Aureobasidium*, and most of these taxa were correlated with the Cd concentration in rice grains (Figure 7b). Most of these fungi were the dominant taxa in the rice rhizosphere (Figure 3a). These fungi may possess HM resistance and alter HM accumulation in plants by coordinating multiple plant processes, including HM sequestration and efflux, the formation of Fe plaques in the rhizosphere, the expressions of Cd transporters, the synthesis of organic acids and others [70,71]. For example, *Acremonium* displayed a high sorption and resistance to HMs (Zn, Cd, etc.) by synthesizing compounds with metal ion-chelating moieties and was positively correlated with host biomass, Cd and Zn concentrations [72,73]. Moreover, *Clonostachys*, *Talaromyces* and *Fusarium* isolates were reported as Cd-resistant fungi and were able to change the available Cd in soil and Cd accumulation in plants [74,75,76,77].

### 4.3. Impact of Soil Factors and Grain Cd Concentration on Rice Seed Endophytic Fungi Community and Its Potential Role in Rice Grain Cd Accumulation

The present study demonstrated that the community of rice SEF was closely related to rhizospheric soil factors and grain Cd concentrations (Figure 7c, Table 2 and Table 3). Similarly, Zhang et al. [78] reported that the stem Cd concentration, soil-available Cd concentration and soil pH were the driving factors affecting the endophyte community of *Sedum plumbizincicola*, and endophyte metabolic pathways were enriched in amino acid metabolism and secondary metabolite metabolism, associated with HM tolerance and accumulation in plants. Contrary to RF, the species diversity and richness of SEF were positively associated with soil nutrients, while being negatively associated with the soil-available Cd concentration and grain Cd concentration (Table 2). In this study, the reads and OTUs of SEF were lower than those of rhizospheric fungi, while fewer fungal genera were enriched in rice seeds from site SC2, with the highest available Cd concentration (Figure 2b and Figure 6b). The reasons may be that endophytic microbes exhibit lower abundance and diversity than rhizospheric microbes due to limited living space and nutrition. Thus, the host plant retains endophytic microbes with more benefits under Cd and nutrient stress, thereby reducing species diversity and richness [79,80,81]. In addition, the samples were collected from paddies near mines, and other metals (lead (Pb), zinc (Zn), etc.) may also influence the microbiome structure. For example, Ma et al. [82] found that, under different HM (Pb, Zn and Cd) stresses, the alpha diversity of endophytic fungi of *Symphytum officinale* decreased; however, the HM-tolerant endophytes were enriched. Furthermore, Peng et al. [83] reported that the species diversity and richness of RF were decreased with the soil AK increase, but the opposite was observed for endophytic fungi.

Moreover, the grain Cd concentration was positively associated with unclassified Pleosporales but negatively associated with unclassified Xylariales (Figure 7d). Sim et al. [84] found that endophytic Pleosporales isolates from *Phragmites* displayed a higher Cd tolerance (>200 mg/L) than that of the Xylariales isolate (100–200 mg/L). Similarly, Soares et al. [85] isolated many endophytic Pleosporales and Xylariales from *Phragmites australis*, and these fungi showed a high tolerance to mercury (Hg) and Zn. These results indicate that rice seeds may be colonized by new endophytic fungal species, which may play an important role in the Cd resistance and grain Cd accumulation of rice and need more attention in future studies. However, due to the similarity of the ITS gene amplicon sequences, the short-read approach often limits the microbial composition analysis at lower levels, such as at the genus or species level. Further studies may be performed with high-precision community analysis, using full-length sequencing and other methods [86].

The previous studies have shown that endophytes mainly derive from the soil and then enter the seed via vascular connections. Over time, the plant may form mutualistic associations with seed endophytes and transmit them vertically to their offspring, and the endophytes may play a crucial role in the health, growth, stress tolerance and productivity of plants [87,88].

## 5. Conclusions

The present study indicated that soil-available nutrients were the key chemical factor affecting Cd accumulation in rice grains. In addition, Cd stress triggered a niche-specific response in rice fungal diversity. The RF diversity (Shannon index) increased with the soil-available Cd concentration, while the diversity and richness (Chao1 index) of SEF decreased with the soil-available Cd concentration. Soil nutrients and the available Cd concentration were the main factors shaping the RF community. Both soil physiochemical properties and grain Cd concentrations drove the rice SEF community. Moreover, the Cd concentration in rice grains was related to some dominant RF and SEF, such as *Talaromyces*, *Fusarium*, *Pseudeurotium*, unclassified Pleosporales, etc. This study offers potential microbial targets for safe food production in Cd-contaminated soil. In the future, these fungi with potential applications should be isolated, and their actual role and mechanism in Cd accumulation in rice grains should be investigated.

## Figures and Tables

**Table 1:** The physiochemical properties of soil and Cd concentration in rice grains from moderately and seriously Cd-contaminated rice paddies.

Site	Physiochemical Properties of Soil					Cd Concentrations		
	OM (g/kg)	HN (mg/kg)	AP (mg/kg)	AK (mg/kg)	pH	TCd-S (mg/kg)	ACd-S (mg/kg)	TCd-G (mg/kg)
MC	82.19 ± 4.48 a	240.03 ± 4.14 a	37.10 ± 0.57 a	137.00 ± 1.83 a	6.72 ± 0.20 a	2.00 ± 0.10 c	1.26 ± 0.08 c	0.13 ± 0.03 b
SC1	43.96 ± 0.87 b	181.10 ± 9.69 b	20.40 ± 1.27 b	116.50 ± 2.89 b	6.81 ± 0.21 a	3.29 ± 0.13 a	2.12 ± 0.09 b	0.16 ± 0.04 b
SC2	45.27 ± 5.34 b	166.55 ± 6.84 c	4.75 ± 0.84 c	89.75 ± 2.21 c	4.68 ± 0.03 b	2.93 ± 0.15 b	2.73 ± 0.22 a	2.58 ± 0.20 a

Note: Different letters mean significant differences among different sites (*p* < 0.05). OM: organic matter; HN: hydrolyzable nitrogen; AP: available phosphorus; AK: available potassium; TCd-S: total cadmium concentration in soil; ACd-S: available cadmium concentration in soil; TCd-G: total cadmium concentration in rice grains. The risk screening values of cadmium are 0.3 mg/kg (pH ≤ 5.5) and 0.6 mg/kg (6.5 < pH ≤ 7.5); the risk intervention values of cadmium are 1.5 mg/kg (pH ≤ 5.5) and 3.0 mg/kg (6.5 < pH ≤ 7.5) [44]. The maximum allowable concentration of cadmium in rice grains of China is 0.2 mg/kg [45]. MC represents the moderately cadmium-contaminated site; SC1 and SC2 represent the severely cadmium-contaminated sites.

**Table 2:** Spearman correlation analysis between environmental factors and fungal alpha diversity indexes.

Factors	Rhizospheric Fungi				Seed Endophytic Fungi			
	Shannon		Chao1		Shannon		Chao1	
	*r*	*p*	*r*	*p*	*r*	*p*	*r*	*p*
OM	−0.4133	0.037 *	0.1189	0.628	0.6084	0.008 **	0.4126	0.152
HN	−0.5674	0.021 *	0.0699	0.774	0.6993	0.006 **	0.6923	0.070
AP	−0.7018	0.009 **	0.0841	0.776	0.6970	0.003 **	0.8021	0.029 *
AK	−0.8932	0.005 **	−0.0909	0.717	0.6643	0.005 **	0.6573	0.035 *
pH	−0.5378	0.044 *	0.3579	0.225	0.3158	0.060	0.4351	0.058
TCd-S	0.3958	0.069	0.4336	0.295	−0.5524	0.018 *	−0.1678	0.366
ACd-S	0.7391	0.016 *	−0.0280	0.920	−0.7622	0.005 **	−0.8042	0.027 *
TCd-G	NA	NA	NA	NA	−0.5149	0.049 *	−0.4623	0.024 *

Note: OM: organic matter; HN: hydrolyzable nitrogen; AP: available phosphorus; AK: available potassium; TCd-S: total cadmium concentration in soil; ACd-S: available cadmium concentration in soil; TCd-G: total cadmium concentration in rice grains. The “NA” represents no value. The “*” indicates statistically significant difference (*, *p* < 0.05; **, *p* < 0.01).

**Table 3:** Component extracted matrix of RDA/CCA for rice rhizospheric fungi and seed endophytic fungi communities.

	Rhizospheric Fungi Community				Seed Endophytic Fungi Community			
	RDA1	RDA2	*r* ^2^	*p*	CCA1	CCA2	*r* ^2^	*p*
OM	−0.1739	−0.9848	0.7506	0.007 **	−0.0541	−0.9985	0.5838	0.037 *
HN	−0.0497	−0.9988	0.8145	0.003 **	0.1539	−0.9881	0.6031	0.030 *
AP	0.2851	−0.9585	0.8989	0.001 ***	0.5061	−0.8625	0.7685	0.001 ***
AK	0.3085	−0.9512	0.9012	0.001 ***	0.5295	−0.8483	0.7606	0.002 **
pH	0.6516	−0.7586	0.8029	0.003 **	0.8527	−0.5225	0.7708	0.004 **
TCd-S	0.4244	0.9055	0.7030	0.009 **	0.3217	0.9469	0.6346	0.019 *
ACd-S	−0.2263	0.9741	0.8521	0.002 **	−0.4183	0.9083	0.6526	0.013 *
TCd-G	NA	NA	NA	NA	−0.8321	0.5547	0.7604	0.004 **

Note: OM: organic matter; HN: hydrolyzable nitrogen; AP: available phosphorus; AK: available potassium; TCd-S: total cadmium concentration in soil; ACd-S: available cadmium concentration in soil; TCd-G: total cadmium concentration in rice grains. The “NA” represents no value. The “*” indicates statistically significant difference (*, *p* < 0.05; **, *p* < 0.01; ***, *p* < 0.001).

## Data Availability

The raw data generated during the current study are deposited in the Sequence Read Archive (SRA) of the National Center for Biotechnology Information (NCBI) under accession number PRJNA1347199.

## References

[1] Azhar U., Ahmad H., Shafqat H., Babar M., Shahzad Munir H.M., Sagir M., Arif M., Hassan A., Rachmadona N., Rajendran S. (2022). Remediation Techniques for Elimination of Heavy Metal Pollutants from Soil: A Review. Environ. Res..

[2] Zhang Y., Zhang Q., Chen W., Shi W., Cui Y., Chen L., Shao J. (2023). Source Apportionment and Migration Characteristics of Heavy Metal(Loid)s in Soil and Groundwater of Contaminated Site. Environ. Pollut..

[3] Imseng M., Wiggenhauser M., Keller A., Müller M., Rehkämper M., Murphy K., Kreissig K., Frossard E., Wilcke W., Bigalke M. (2018). Fate of Cd in Agricultural Soils: A Stable Isotope Approach to Anthropogenic Impact, Soil Formation, and Soil-Plant Cycling. Environ. Sci. Technol..

[4] Yuan X., Xue N., Han Z. (2021). A Meta-Analysis of Heavy Metals Pollution in Farmland and Urban Soils in China over the Past 20 Years. J. Environ. Sci..

[5] Kumar P., Alhag S.K., Al-Shahari E.A., Al-Fakeh M.S., Abou Fayssal S., Bachheti R.K., Širić I., Eid E.M. (2024). Impact of Irrigation with Contaminated River Water on Growth, Yield, and Heavy Metals Accumulation in Planted Armenian Cucumber (*Cucumis melo* var. *flexuosus* (L.) Naudin.). Water Air Soil. Pollut..

[6] Abou Fayssal S., Kumar P., Popescu S.M., Khanday M.U.D., Sardar H., Ahmad R., Gupta D., Kumar Gaur S., Alharby H.F., Al-Ghamdi A.G. (2024). Health Risk Assessment of Heavy Metals in Saffron (*Crocus sativus* L.) Cultivated in Domestic Wastewater and Lake Water Irrigated Soils. Heliyon.

[7] Huybrechts M., Hendrix S., Kyndt T., Demeestere K., Vandamme D., Cuypers A. (2021). Short-Term Effects of Cadmium on Leaf Growth and Nutrient Transport in Rice Plants. Plant Sci..

[8] Huang J.-L., Li Z.-Y., Mao J.-Y., Chen Z.-M., Liu H.-L., Liang G.-Y., Zhang D.-B., Wen P.-J., Mo Z.-Y., Jiang Y.-M. (2024). Contamination and Health Risks Brought by Arsenic, Lead and Cadmium in a Water-Soil-Plant System Nearby a Non-Ferrous Metal Mining Area. Ecotoxicol. Environ. Saf..

[9] Satarug S., Vesey D.A., Gobe G.C., Phelps K.R. (2023). Estimation of Health Risks Associated with Dietary Cadmium Exposure. Arch. Toxicol..

[10] Rigby H., Smith S.R. (2020). The Significance of Cadmium Entering the Human Food Chain via Livestock Ingestion from the Agricultural Use of Biosolids, with Special Reference to the UK. Environ. Int..

[11] Zhang J., Liu Y.-X., Zhang N., Hu B., Jin T., Xu H., Qin Y., Yan P., Zhang X., Guo X. (2019). *NRT1.1B* Is Associated with Root Microbiota Composition and Nitrogen Use in Field-Grown Rice. Nat. Biotechnol..

[12] Liu Q., Cheng L., Nian H., Jin J., Lian T. (2023). Linking Plant Functional Genes to Rhizosphere Microbes: A Review. Plant Biotechnol. J..

[13] He X., Xiao X., Wei W., Li L., Zhao Y., Zhang N., Wang M. (2024). Soil Rare Microorganisms Mediated the Plant Cadmium Uptake: The Central Role of Protists. Sci. Total Environ..

[14] Altaf M., Ilyas T., Shahid M., Shafi Z., Tyagi A., Ali S. (2024). *Trichoderma* Inoculation Alleviates Cd and Pb-Induced Toxicity and Improves Growth and Physiology of *Vigna radiata* (L.). ACS Omega.

[15] Hussain I., Irshad M., Hussain A., Qadir M., Mehmood A., Rahman M., Alrefaei A.F., Almutairi M.H., Ali S., Hamayun M. (2024). Phosphate Solubilizing *Aspergillus Niger* PH1 Ameliorates Growth and Alleviates Lead Stress in Maize through Improved Photosynthetic and Antioxidant Response. BMC Plant Biol..

[16] Zhou X., Zhang X., Ma C., Wu F., Jin X., Dini-Andreote F., Wei Z. (2022). Biochar Amendment Reduces Cadmium Uptake by Stimulating Cadmium-Resistant PGPR in Tomato Rhizosphere. Chemosphere.

[17] Duan R., Zhang Y., Dai Q., Yang L., Yang H., Meng F., Hu W., Zhang P. (2025). *Phanerochaete chrysosporium* Reduces Heavy Metal Uptake in Rice by Affecting Rhizosphere Microbes and Root Metabolism. Ecotoxicol. Environ. Saf..

[18] Luo C., Li T., Huang Y., Liu T., Dong Y. (2025). Exogenous Nano-Silicon Enhances the Ability of Intercropped Faba Bean to Alleviate Cadmium Toxicity and Resist *Fusarium wilt*. J. Nanobiotechnol..

[19] Sharma N., Yadav G., Tyagi J., Kumar A., Koul M., Joshi N.C., Hashem A., Abd Allah E.F., Mishra A. (2024). Synergistic Impact of Serendipita Indica and *Zhihengliuella* sp. ISTPL4 on the Mitigation of Arsenic Stress in Rice. Front. Microbiol..

[20] Wu M., Tang T., Liu Q., Xiao P., Xiong Y., Feng J., Peng M., Zhu J., Yong X., Jia Y. (2025). Characterization of Endophytic Bacterial Communities in *Abelmoschus Manihot* under Cd Stress and Isolation of Cd-Resistant Bacteria. J. Hazard. Mater..

[21] Lin Z., Qiao Y., Xu K., Lu L., Shu Q., Tian S. (2025). The Endophytic Fungus *Serendipita Indica* Reshapes Rhizosphere Soil Microbiota to Improve *Salix suchowensis* Growth and Phytoremediation. J. Hazard. Mater..

[22] Durand A., Leglize P., Lopez S., Sterckeman T., Benizri E. (2022). *Noccaea Caerulescens* Seed Endosphere: A Habitat for an Endophytic Bacterial Community Preserved through Generations and Protected from Soil Influence. Plant Soil..

[23] Sharma V.K., Parmar S., Tang W., Hu H., White J.F., Li H. (2022). Effects of Fungal Seed Endophyte FXZ2 on *Dysphania ambrosioides* Zn/Cd Tolerance and Accumulation. Front. Microbiol..

[24] Truyens S., Jambon I., Croes S., Janssen J., Weyens N., Mench M., Carleer R., Cuypers A., Vangronsveld J. (2014). The Effect of Long-Term Cd and Ni Exposure on Seed Endophytes of *Agrostis capillaris* and Their Potential Application in Phytoremediation of Metal-Contaminated Soils. Int. J. Phytoremediat..

[25] Song J., Song Q., Wang D., Liu Y. (2023). Mitigation Strategies for Excessive Cadmium in Rice. Compr. Rev. Food Sci. Food Saf..

[26] Xie L.H., Tang S.Q., Wei X.J., Shao G.N., Jiao G.A., Sheng Z.H., Luo J., Hu P.S. (2017). The Cadmium and Lead Content of the Grain Produced by Leading Chinese Rice Cultivars. Food Chem..

[27] Shi Z., Carey M., Meharg C., Williams P.N., Signes-Pastor A.J., Triwardhani E.A., Pandiangan F.I., Campbell K., Elliott C., Marwa E.M. (2020). Rice Grain Cadmium Concentrations in the Global Supply-Chain. Expo. Health.

[28] Li H., Luo N., Li Y.W., Cai Q.Y., Li H.Y., Mo C.H., Wong M.H. (2017). Cadmium in Rice: Transport Mechanisms, Influencing Factors, and Minimizing Measures. Environ. Pollut..

[29] Zou M., Zhou S., Zhou Y., Jia Z., Guo T., Wang J. (2021). Cadmium Pollution of Soil-Rice Ecosystems in Rice Cultivation Dominated Regions in China: A Review. Environ. Pollut..

[30] Wang R., Xiao Y., Lv F., Hu L., Wei L., Yuan Z., Lin H. (2018). Bacterial Community Structure and Functional Potential of Rhizosphere Soils as Influenced by Nitrogen Addition and Bacterial Wilt Disease under Continuous Sesame Cropping. Appl. Soil Ecol..

[31] Radhakrishnan N.A., Ravi A., Joseph B.J., Jose A., Jithesh O., Krishnankutty R.E. (2023). Phenazine 1-Carboxylic Acid Producing Seed Harbored Endophytic Bacteria from Cultivated Rice Variety of Kerala and Its Broad Range Antagonism to Diverse Plant Pathogens. Probiotics Antimicrob. Proteins.

[32] Wang Y., Li P., Tian Y., Xiong Z., Zheng Z., Yi Z., Ao H., Wang Q., Li J. (2023). Bacterial Seed Endophyte and Abiotic Factors Influence Cadmium Accumulation in Rice (*Oryza sativa*) along the Yangtze River Area. Ecotoxicol. Environ. Saf..

[33] Pan X., Zhang S., Zhong Q., Gong G., Wang G., Guo X., Xu X. (2020). Effects of Soil Chemical Properties and Fractions of Pb, Cd, and Zn on Bacterial and Fungal Communities. Sci. Total Environ..

[34] Parmar S., Li Q., Wu Y., Li X., Yan J., Sharma V.K., Wei Y., Li H. (2018). Endophytic Fungal Community of *Dysphania ambrosioides* from Two Heavy Metal-Contaminated Sites: Evaluated by Culture-Dependent and Culture-Independent Approaches. Microb. Biotechnol..

[35] Zhang F., Peng D., Liu L., Jiang H., Bai L. (2022). Cultivar-Dependent Rhizobacteria Community and Cadmium Accumulation in Rice: Effects on Cadmium Availability in Soils and Iron-Plaque Formation. J. Environ. Sci..

[36] Ren Z., Chen A.J., Zong Q., Du Z., Guo Q., Liu T., Chen W., Gao L. (2023). Microbiome Signature of Endophytes in Wheat Seed Response to Wheat Dwarf Bunt Caused by Tilletia Controversa Kühn. Microbiol. Spectr..

[37] Ma Y., Gao W., Zhang F., Zhu X., Kong W., Niu S., Gao K., Yang H. (2022). Community Composition and Trophic Mode Diversity of Fungi Associated with Fruiting Body of Medicinal *Sanghuangporus vaninii*. BMC Microbiol..

[38] Costa D., Fernandes T., Martins F., Pereira J.A., Tavares R.M., Santos P.M., Baptista P., Lino-Neto T. (2021). Illuminating *Olea europaea* L. Endophyte Fungal Community. Microbiol. Res..

[39] Li C., Wu Y., Li L., Zhao C., Li B., Wu Y., Wang H., Yan Z. (2023). Different Techniques Reveal the Difference of Community Structure and Function of Fungi from Root and Rhizosphere of *Salvia miltiorrhiza* Bunge. Plant Biol..

[40] Liu J., Wang Z., Chen Z., White J.F., Malik K., Chen T., Li C. (2022). Inoculation of Barley (*Hordeum vulgare*) with the Endophyte *Epichloë bromicola* Affects Plant Growth, and the Microbial Community in Roots and Rhizosphere Soil. J. Fungi.

[41] Ren G., Zhang H., Lin X., Zhu J., Jia Z. (2015). Response of Leaf Endophytic Bacterial Community to Elevated CO_2_ at Different Growth Stages of Rice Plant. Front. Microbiol..

[42] Cestarić D., Škvorc Ž., Franjić J., Sever K., Krstonošić D. (2017). Forest Plant Community Changes in the Spačva Lowland Area (E Croatia). Plant Biosyst. Int. J. Deal. All Asp. Plant Biol..

[43] Sun Y., Xu Y., Zhang J., Bello A., Li X., Liu W., Egbeagu U.U., Zhao L., Han Y., Cheng L. (2023). Investigation of Underlying Links between Nitrogen Transformation and Microorganisms’ Network Modularity in the Novel Static Aerobic Composting of Dairy Manure by “Stepwise Verification Interaction Analysis”. Sci. Total Environ..

[44] (2018). Soil Environmental Quality-Risk Control Standard for Soil Contamination of Agricultural Land.

[45] (2022). National Food Safety Standard-Maximum Levels of Contaminants in Foods.

[46] Chen B., Tan S., Zeng Q., Wang A., Zheng H. (2019). Soil Nutrient Heterogeneity Affects the Accumulation and Transfer of Cadmium in Bermuda Grass (*Cynodon dactylon* (L.) Pers.). Chemosphere.

[47] Yang Y., Xiong J., Tao L., Cao Z., Tang W., Zhang J., Yu X., Fu G., Zhang X., Lu Y. (2020). Regulatory Mechanisms of Nitrogen (N) on Cadmium (Cd) Uptake and Accumulation in Plants: A Review. Sci. Total Environ..

[48] Shao X., Yao H., Cui S., Peng Y., Gao X., Yuan C., Chen X., Hu Y., Mao X. (2021). Activated Low-Grade Phosphate Rocks for Simultaneously Reducing the Phosphorus Loss and Cadmium Uptake by Rice in Paddy Soil. Sci. Total Environ..

[49] Huang B., Liao Q., Fu H., Ye Z., Mao Y., Luo J., Wang Y., Yuan H., Xin J. (2023). Effect of Potassium Intake on Cadmium Transporters and Root Cell Wall Biosynthesis in Sweet Potato. Ecotoxicol. Environ. Saf..

[50] Wang K., Fu G., Yu Y., Wan Y., Liu Z., Wang Q., Zhang J., Li H. (2019). Effects of Different Potassium Fertilizers on Cadmium Uptake by Three Crops. Environ. Sci. Pollut. Res. Int..

[51] Borgo L., Rabêlo F.H.S., Rossi M.L., dos Santos F.H., Nogueira M.L.G., Alleoni L.R.F., Linhares F.S., Vangronsveld J., Lavres J. (2023). Effect of Selenium and Soil pH on Cadmium Phytoextraction by *Urochloa decumbens* Grown in Oxisol. J. Hazard. Mater..

[52] Wei B., Peng Y., Jeyakumar P., Lin L., Zhang D., Yang M., Zhu J., Ki Lin C.S., Wang H., Wang Z. (2023). Soil pH Restricts the Ability of Biochar to Passivate Cadmium: A Meta-Analysis. Environ. Res..

[53] Xie L., Li W., Pang X., Liu Q., Yin C. (2023). Soil Properties and Root Traits Are Important Factors Driving Rhizosphere Soil Bacterial and Fungal Community Variations in Alpine *Rhododendron nitidulum* Shrub Ecosystems along an Altitudinal Gradient. Sci. Total Environ..

[54] Chaudhary P., Xu M., Ahamad L., Chaudhary A., Kumar G., Adeleke B.S., Verma K.K., Hu D.-M., Širić I., Kumar P. (2023). Application of Synthetic Consortia for Improvement of Soil Fertility, Pollution Remediation, and Agricultural Productivity: A Review. Agronomy.

[55] Naz M., Dai Z., Hussain S., Tariq M., Danish S., Khan I.U., Qi S., Du D. (2022). The Soil pH and Heavy Metals Revealed Their Impact on Soil Microbial Community. J. Environ. Manag..

[56] Xiong R., He X., Gao N., Li Q., Qiu Z., Hou Y., Shen W. (2024). Soil pH Amendment Alters the Abundance, Diversity, and Composition of Microbial Communities in Two Contrasting Agricultural Soils. Microbiol. Spectr..

[57] Xu P., Gao M., Li Y., Ye J., Su J., Li H. (2025). Combined Effects of Acidification and Warming on Soil Denitrification and Microbial Community. Front. Microbiol..

[58] Sun R., Zhang W., Liu Y., Yun W., Luo B., Chai R., Zhang C., Xiang X., Su X. (2022). Changes in Phosphorus Mobilization and Community Assembly of Bacterial and Fungal Communities in Rice Rhizosphere under Phosphate Deficiency. Front. Microbiol..

[59] Zapata D., López J.E., Saldarriaga J.F. (2024). Plant Growth-Promoting and Biocontrol Potential of *Aspergillus tubingensis* and *Talaromyces islandicus*. J. Soil. Sci. Plant Nutr..

[60] Long X.-E., Yao H. (2020). Phosphorus Input Alters the Assembly of Rice (*Oryza sativa* L.) Root-Associated Communities. Microb. Ecol..

[61] Li Q., Wu Q., Zhang T., Xiang P., Bao Z., Tu W., Li L., Wang Q. (2022). Phosphate Mining Activities Affect Crop Rhizosphere Fungal Communities. Sci. Total Environ..

[62] Ma B., Song W., Zhang X., Chen M., Li J., Yang X., Zhang L. (2023). Potential Application of Novel Cadmium-Tolerant Bacteria in Bioremediation of Cd-Contaminated Soil. Ecotoxicol. Environ. Saf..

[63] Philippot L., Chenu C., Kappler A., Rillig M.C., Fierer N. (2024). The Interplay between Microbial Communities and Soil Properties. Nat. Rev. Microbiol..

[64] Xu Y., Seshadri B., Bolan N., Sarkar B., Ok Y.S., Zhang W., Rumpel C., Sparks D., Farrell M., Hall T. (2019). Microbial Functional Diversity and Carbon Use Feedback in Soils as Affected by Heavy Metals. Environ. Int..

[65] Lin Y., Xiao W., Ye Y., Wu C., Hu Y., Shi H. (2020). Adaptation of Soil Fungi to Heavy Metal Contamination in Paddy Fields-a Case Study in Eastern China. Environ. Sci. Pollut. Res. Int..

[66] Liu H., Yao J., Liu B., Li M., Liu J., Jiang S., Yu W., Zhao Y., Duran R. (2023). Active Tailings Disturb the Surrounding Vegetation Soil Fungal Community: Diversity, Assembly Process and Co-Occurrence Patterns. Sci. Total Environ..

[67] Ważny R., Jędrzejczyk R.J., Domka A., Pliszko A., Kosowicz W., Githae D., Rozpądek P. (2023). How Does Metal Soil Pollution Change the Plant Mycobiome?. Environ. Microbiol..

[68] Song J., Shen Q., Wang L., Qiu G., Shi J., Xu J., Brookes P.C., Liu X. (2018). Effects of Cd, Cu, Zn and Their Combined Action on Microbial Biomass and Bacterial Community Structure. Environ. Pollut..

[69] Luo Z., Wu S., Shi W., Hu H., Lin T., Zhao K., Hou G., Fan C., Li X., Chen G. (2024). Combined Effects of Cadmium and Simulated Acid Rain on Soil Microbial Communities in the Early Cultivation of *Populus beijingensis* Seedlings. Ecotoxicol. Environ. Saf..

[70] Priyanka, Dwivedi S.K. (2023). Fungi Mediated Detoxification of Heavy Metals: Insights on Mechanisms, Factors and Recent. J. Water Process Eng..

[71] Dilawar N., Hamayun M., Iqbal A., Lee B., Ali S., Ahmad A., Alrefaei A.F., Faraj T.K., Kim H.-Y., Hussain A. (2024). Rhizofungus *Aspergillus terreus* Mitigates Heavy Metal Stress-Associated Damage in *Triticum aestivum* L. Plants.

[72] Mohammadian E., Babai Ahari A., Arzanlou M., Oustan S., Khazaei S.H. (2017). Tolerance to Heavy Metals in Filamentous Fungi Isolated from Contaminated Mining Soils in the Zanjan Province, Iran. Chemosphere.

[73] Jiang Y., Luo J., Guo X., Qiao Y., Li Y., Zhang Y., Zhou R., Vaculík M., Li T. (2025). Phyllosphere Microbiome Assists the Hyperaccumulating Plant in Resisting Heavy Metal Stress. J. Environ. Sci..

[74] Shadmani L., Jamali S., Fatemi A. (2021). Isolation, Identification, and Characterization of Cadmium-Tolerant Endophytic Fungi Isolated from Barley (*Hordeum vulgare* L.) Roots and Their Role in Enhancing Phytoremediation. Braz. J. Microbiol..

[75] Wang S., Dai H., Wei S., Skuza L., Chen Y. (2022). Effects of Cd-Resistant Fungi on Uptake and Translocation of Cd by Soybean Seedlings. Chemosphere.

[76] Xiao Y., Chen R., Chen L., Yang B., Jiang L., Fang J. (2023). Endophytic Fungus *Talaromyces* sp. MR1 Promotes the Growth and Cadmium Uptake of *Arabidopsis thaliana* L. Under Cadmium Stress. Curr. Microbiol..

[77] Gu T., Qi Z., Wang Y., Chen S., Yan J., Qiu H., Yu Y., Fang Z., Wang J., Gong J. (2024). An Endophytic Fungus Interacts with the Defensin-like Protein OsCAL1 to Regulate Cadmium Allocation in Rice. Mol. Plant.

[78] Zhang J., Na M., Wang Y., Ge W., Zhou J., Zhou S. (2024). Cadmium Levels and Soil pH Drive Structure and Function Differentiation of Endophytic Bacterial Communities in *Sedum plumbizincicola*: A Field Study. Sci. Total Environ..

[79] Tan Y., Cui Y., Li H., Kuang A., Li X., Wei Y., Ji X. (2017). Rhizospheric Soil and Root Endogenous Fungal Diversity and Composition in Response to Continuous *Panax notoginseng* Cropping Practices. Microbiol. Res..

[80] Wang L., Gong L., Gan D., Li X., Yao J., Wang L., Qu J., Cong J., Zhang Y. (2022). Diversity, Function and Assembly of the *Trifolium repens* L. Root-Associated Microbiome under Lead Stress. J. Hazard. Mater..

[81] Zhang F., Peng R., Xie Y., Ji X., Liu S., Jiang H. (2025). Cultivar-Specific Response of a Root-Associated Microbiome Assembly of Rice to Cadmium Pollution. Plant Physiol. Biochem..

[82] Ma J., Chen D., Xu Y., Liu Y., Liu L., Huang J., Gao R., Bai J., Hou Q. (2024). Effects of Different Heavy Metal Stressors on the Endophytic Community Composition and Diversity of *Symphytum officinale*. Microorganisms.

[83] Peng M., He H., Wang X., Wang Z., Zhuang L. (2023). Comparison of Network Connectivity and Environmental Driving Factors of Root-Associated Fungal Communities of Desert Ephemeral Plants in Two Habitat Soils. J. Environ. Manag..

[84] Sim C.S.F., Cheow Y.L., Ng S.L., Ting A.S.Y. (2018). Discovering Metal-Tolerant Endophytic Fungi from the Phytoremediator Plant Phragmites. Water Air Soil. Pollut..

[85] Soares M.A., Li H.-Y., Kowalski K.P., Bergen M., Torres M.S., White J.F. (2016). Evaluation of the Functional Roles of Fungal Endophytes of *Phragmites australis* from High Saline and Low Saline Habitats. Biol. Invasions.

[86] Yan K., Zhou J., Feng C., Wang S., Haegeman B., Zhang W., Chen J., Zhao S., Zhou J., Xu J. (2023). Abundant Fungi Dominate the Complexity of Microbial Networks in Soil of Contaminated Site: High-Precision Community Analysis by Full-Length Sequencing. Sci. Total Environ..

[87] War A.F., Bashir I., Reshi Z.A., Kardol P., Rashid I. (2023). Insights into the Seed Microbiome and Its Ecological Significance in Plant Life. Microbiol. Res..

[88] Abdelfattah A., Tack A.J.M., Lobato C., Wassermann B., Berg G. (2023). From Seed to Seed: The Role of Microbial Inheritance in the Assembly of the Plant Microbiome. Trends Microbiol..

